# Recent Trends in Curcumin-Containing Inorganic-Based Nanoparticles Intended for In Vivo Cancer Therapy

**DOI:** 10.3390/pharmaceutics16020177

**Published:** 2024-01-26

**Authors:** Douglas Dourado, Júlio Abreu Miranda, Matheus Cardoso de Oliveira, Danielle Teixeira Freire, Francisco Humberto Xavier-Júnior, Edgar Julian Paredes-Gamero, Éverton do Nascimento Alencar

**Affiliations:** 1 Department of Immunology, Aggeu Magalhães Institute (IAM), Oswaldo Cruz Foundation (FIOCRUZ), Recife 50670-420, PE, Brazil; ddourado.science@gmail.com; 2 Department of Pharmacy, Federal University of Rio Grande do Norte (UFRN), Natal 59010-180, RN, Brazil; julioabreumiranda@gmail.com (J.A.M.); matheuscardoso-oliveira@outlook.com (M.C.d.O.); 3 College of Pharmaceutical Sciences, Food and Nutrition, Federal University of Mato Grosso do Sul (UFMS), Campo Grande 79070-900, MS, Brazil; danielletfreire@gmail.com (D.T.F.); edgar-gamero@ufms.br (E.J.P.-G.); 4 Laboratory of Pharmaceutical Biotechnology (BioTecFarm), Department of Pharmacy, Federal University of Paraíba (UFPB), João Pessoa 58051-900, PB, Brazil; fhxj@academico.ufpb.br

**Keywords:** curcuminoids, nanotechnology, pre-clinical, oncology

## Abstract

Curcumin is a natural compound that has been widely investigated thanks to its various biological properties, including antiproliferative. This molecule acts on different cancers such as lung, breast, pancreatic, colorectal, etc. However, the bioactive actions of curcumin have limitations when its physicochemical properties compromise its pharmacological potential. As a therapeutic strategy against cancer, curcumin has been associated with inorganic nanoparticles. These nanocarriers are capable of delivering curcumin and offering physicochemical properties that synergistically enhance anticancer properties. This review highlights the different types of curcumin-based inorganic nanoparticles and discusses their physicochemical properties and in vivo anticancer activity in different models of cancer.

## 1. Introduction

Cancer is categorized as a leading cause of mortality worldwide [1]. It is a pathological state that results from a build-up of genetic abnormalities and is characterized by the unchecked growth, proliferation, and differentiation of cells [2]. Current treatments are based on curative, reconstructive, and/or palliative care [3,4]. Notwithstanding the progress made in the last few decades, the treatment of cancer might be resistant to traditional chemotherapeutic agents. Hence, new targeted drugs remain a significant endeavor in cancer therapy [5]. Recently, there has been a greater focus on employing natural chemicals in chemotherapy to raise the therapeutic index of certain anticancer molecules and to act as a complementary treatment [6]. In this context, curcumin emerges as a potential bioactive with extensive anticancer potential against different types of cancer [7]. Curcumin is a major natural compound obtained from the rhizome of *Curcuma longa* L. [8]. Despite its high biological potential, it has biopharmaceutical limitations that compromise its clinical use in cancer therapy [9].

Nanoparticles are a nanotechnology tool that has been successfully applied in delivering anticancer molecules with biopharmaceutical limitations [10,11,12]. These can be used to treat cancer due to their specific advantages such as biocompatibility, reduced toxicity, enhanced permeability and retention (EPR) effect, and precise targeting [13,14]. In this perspective, different nanoparticles of organic nature (i.e., polymeric and lipid) for curcumin delivery were developed as anticancer therapeutic alternatives [15,16,17].

Other nanoparticles for curcumin delivery that deserve greater attention are inorganic nanoparticles. These are nanocarriers made of metallic structures, metallic oxides, among others [18]. These nanoparticles are more advantageous than those of an organic nature when it comes to their optimized physicochemical properties, which make a difference in cancer therapy [19]. They can successfully carry curcumin and show synergy with this natural compound in cancer therapy [20,21,22]. Although different literature reviews have addressed different nanostructures containing curcumin, there is still a lack of association between curcumin inorganic nanostructures’ physicochemical properties and their in vivo anticancer effects. Therefore, this narrative review aims to highlight recent advances in inorganic nanoparticles used to deliver curcumin and to discuss this drug’s physicochemical and anticancer property in in vivo models.

## 2. Curcumin and Its Anticancer Effects

Different natural compounds obtained from plants have shown anticancer activity. Among these, polyphenols are a class of natural plant chemicals that have demonstrated anticancer properties [23]. These bioactives derive from phenylalanine and contain an aromatic ring with one or more hydroxyl groups. They include a large class of antioxidants such as flavonoids, phenolic acids and their derivatives, lignans, and stilbenes [24]. Numerous mechanisms relate to polyphenols´ anticancer effects, such as the following: (i) removing cancer cells by altering signaling pathways; (ii) inhibiting cell cycle events; (iii) inducing apoptosis; and (iv) regulating the activities of enzymes involved in tumor cell proliferation [25].

In this scenario, we find curcumin, a polyphenol isolated from the rhizome of *Curcuma longa* L. [8]. Its chemical structure consists of two aromatic rings containing hydroxyl and methoxy groups, which are linked through a chain containing seven carbons of an α,β-unsaturated β-diketone moiety [26,27]. This molecule shows high tolerability and safety in physiological environments (i.e., human models) [28,29,30,31], as well as significant biological potential for therapeutic application. Among its potentialities, curcumin acts both in chemoprevention and directly in cancer cells [9,32]. In vitro and in vivo studies have shown the anticancer activity of curcumin against (i) breast, (ii) lung, (iii) prostate, (iv) pancreas, and (v) colorectal cancer, among other types [7,33,34,35].

Curcumin induces apoptosis while inhibiting the proliferation and invasion of tumors by suppressing various cellular signaling pathways. More specifically, curcumin can (i) restrict proliferation, reducing the cell cycle through inhibition of the Wnt/β-catenin pathway, increasing the levels of p53, p21, and p27, and inhibiting the levels of CDK4 and Cyclin D1 [35]. It can (ii) increase E-cadherin levels and decrease N-cadherin, vimentin, fibronectin, slug, and snail levels through suppression of the TGF-β/Smad2/3 pathway, inhibiting migration and invasion [36]. Further, it can (iii) stimulate the production of ROS through the activation of the p38 MAPK, JNK, and ERK pathways. Additionally, curcumin can (iv) set ferroptosis, increasing the levels of TFRC, FTL, and FTH1, and (v) promote apoptosis by enhancing the expression or cleavage of apoptotic proteins (Bax, Cleaved-caspase-3, Cleaved-caspase-9, and Cleaved-PARP) and by inhibiting the expression of anti-apoptotic proteins (Bcl-2) [37]. Curcumin (vi) can enhance the expressions of Beclin1, Atg5, Atg3, and LC3B-II/I and promote autophagy by the PI3K/Akt/mTOR pathway. Furthermore, curcumin can (vii) reduce the levels of Oct4, Sox2, and Nanog suppressing stemness through the inhibition of JAK/STAT3 pathways; (viii) suppress the TLR4/NF-κB signaling pathway attenuating inflammation (TNF-α, IL-6, and IL-1β); and (ix) mitigate angiogenesis through the inhibition of the expressions of VEGF, CD31, αSMC, iNOS, and COX-2 [7,38].

Although curcumin reveals promising properties, it presents critical limitations, such as (i) quick systemic elimination; (ii) significant first-pass intestinal (phase I) and hepatic (phase II) metabolism; (iii) instability in intestinal pH; (iv) poor intestinal permeability; and (v) low water solubility [39,40,41,42]. These aspects have been key in limiting curcumin’s clinical acceptance as a therapeutic agent [43].

One of the strategies to improve its biological efficacy (i.e., anticancer) has been its complexation with metals [44]. The α,β-unsaturated β-diketo moiety of curcumin is reported to be a strong chelating agent; it interacts with various metal ions [45]. Based on these advantageous interactions, developing inorganic-based nanoparticles, whose nanostructures provide important characteristics for increasing the effectiveness of cancer therapy, is key to curcumin delivery [21].

## 3. Curcumin-Based Inorganic Nanoparticles—Physicochemical Characteristics

**Figure 1:**
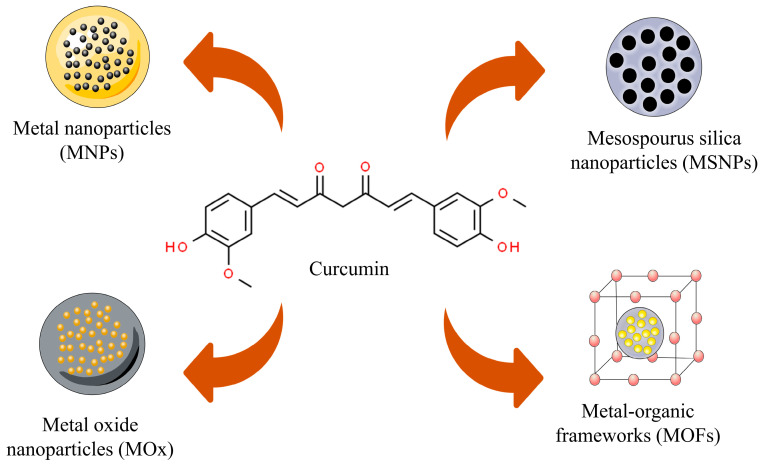
Curcumin-based inorganic nanoparticle types that have been designed and tested in vivo in cancer models. Metal nanoparticles (MNPs), mesoporous silica nanoparticles (MSNPs), metal oxide nanoparticles (MOx), and metal–organic frameworks (MOFs).

Inorganic nanoparticles are nanostructures made of metals, oxide metals, among other structures [46]. These nanostructures have been used in cancer studies due to their ability to target ligands increasing therapeutic impact and reducing off-target side effects through drug adsorption and penetration [21]. In this perspective, different curcumin-based inorganic nanoparticles have been designed and tested using in vivo models. Among such, metal nanoparticles (gold–MNPs), metal oxides (superparamagnetic iron oxide, graphene oxide, and zinc–MOx), mesoporous silica nanoparticles (MSNPs), and metal–organic frameworks (MOFs) can be highlighted (Figure 1).

### 3.1. Metal Nanoparticles

**Figure 2:**
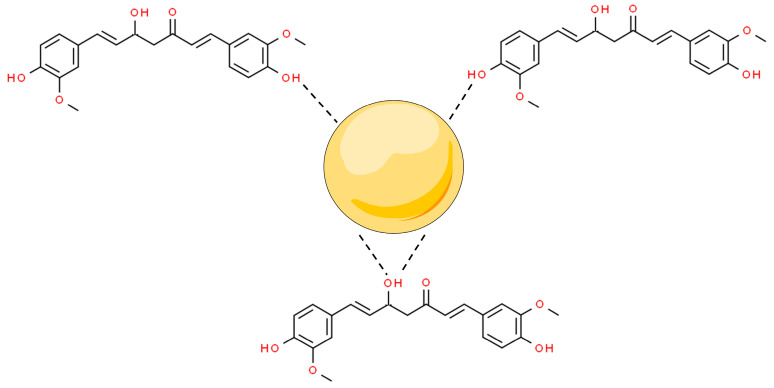
Overall scheme of curcumin hydroxyl from enol and phenol groups’ interaction to metal nanoparticles’ (MNPs’) surfaces. Based on Amaldoss et al., 2022; Wanninger et al., 2015; and Prasad et al., 2021 [21,44,45].

Metal nanoparticles (MNPs) are nanomaterials that are made of one chemical element. They can be synthesized from their metal precursors and modified with several functional groups. This allows them to be conjugated with antibodies, ligands, and drugs of interest presenting a wide range of applications [47,48]. Among their applications, MNPs can modulate (i) apoptosis and (ii) cell cycle arrest; and inhibit (iii) tumor angiogenesis, (iv) metastasis, and (v) inflammation to stop cancer proliferation. Moreover, metal nanoparticles have shown synergistic potential with marketed anticancer drugs, improving their bioactivity and bioavailability [20]. In this scenario, curcumin has been conjugated with MNPs, by capping the surface of the nanostructures mediated by interactions with the hydroxyl moieties of the two phenolic groups and one enolic group from this molecule (Figure 2).

MNPs can be built from different elements. In the biomedical field, gold is extensively used thanks to its advantageous properties. One could mention its high electrical conductivity and reflectivity, low cytotoxicity, biocompatibility, optical properties, malleability, and resistance to corrosion and oxidation. Further, it presents a high degree of size and shape control, besides a relatively bio-inert surface, which can be easily modified [49].

Based on these properties, Mahalunkar and colleagues [50] developed folate–curcumin-loaded gold–polyvinylpyrrolidone nanoparticles (FA–CurAu-PVP NPs). Polyvinylpyrrolidone (PVP) is a non-toxic, non-ionic polymer used in NP synthesis [51]. PVP acts as a surface stabilizer, growth modifier, nanoparticle dispersant, and reducing agent depending on the specific synthetic conditions and material system [52].

These authors [50] obtained FA-CurAu-PVP NPs by LBL (layer-by-layer) assembly. AuNPs, AuPVP NPs, CurAu-PVP NPs, and FA–CurAu-PVP NPs presented hydrodynamic diameters of 36.85 nm, 62.50 nm, 72.56 nm, and 358.7 nm, respectively. The increase in the particle size of AuNPs and AuPVP NPs is related to partial cross-linking between the particles during the conjugation process. The increase in particle size after functionalization with folate (FA) was expected, as reported in the literature [53]. The coating of the nanoparticles with PVP and functionalization with FA were confirmed using UV-Vis by monitoring the wavelength scan at each stage of synthesis. TEM analysis of FA-CurAu-PVP NCs showed structures with discrete spherical outlines and monodisperse size distribution (~250 nm). The droplet size obtained by TEM (solid diameter) was smaller than the hydrodynamic diameter from DLS, indicating a large electrical double layer in suspension. The solid diameter obtained by TEM was ideal for the passive targeting of tumors [50]. All developed nanoparticles showed a negative electrostatic charge below ±30 mV, an ideal range when it comes to the electrostatic stability for nanoparticles [54]. Further, the nanoparticles contain PVP, which prevents the aggregation of NPs by the steric hindrance effect [52].

Alibolandi and colleagues [55] also developed gold nanoparticles; however, they were stabilized by dendrimers (PAMAM) complexed with PEG and functionalized with MUC-1 aptamer. PAMAM dendrimers are well-defined, highly branched, nanoscaled macromolecules with numerous active amine groups on the surface [56]. These compounds are soluble in the aqueous medium, whereas the three-dimensional interior hollow environment could entrap hydrophobic components, including Au-NP and hydrophobic chemotherapeutic agents [57]. Studies report toxicity from PAMAM’s peripheral amino groups [58] that can be overcome by PEGylation [56,59]. These authors incorporated gold nanoparticles into the interior core of the PEGylated dendrimers. Further, curcumin was incorporated into PAMAM forming unbound curcumin and gold dispersions anchored in PAMAM, instead of curcumin–gold bound to each other. The dendrimers were functionalized with MUC-1 aptamers to selectively deliver the therapeutic platform (gold nanoparticles and curcumin) to the tumor tissue. Vectorization was achieved by targeting MUC-1 receptors, which are overexpressed in several tumors [60,61]. The size of the functionalized nanostructures (Apt-PEG-AuPAMAM NPs) was 5.23 ± 4.12 nm with a polydispersity index (PdI) below 0.2 and a zeta potential of 4.6 ± 0.74 mV. When associated with PEG and later with Au, no significant changes were observed [55]. In addition, PEG-AuPAMAM revealed a curcumin encapsulation efficiency of 82%, most likely due to PEGylation [61].

Although we have already discussed the advantages of using gold nanoparticles complexed with curcumin in this review, it is essential to note that there are also significant benefits to employing gold quantum clusters (Au QCs) for curcumin complexation. These materials are notable due to their reduced size and fascinating physical and chemical properties. In this context, Khandewal and collaborators developed curcumin-Au QCs conjugates (C-Au QCs) intending to preserve the anticancer activity of curcumin while minimizing its toxicity [62]. By transmission electron microscopy (TEM), the authors observed that the particles exhibited excellent uniformity in dispersion, with an average size of 2 nm. Furthermore, the Dynamic Light Scattering (DLS) technique revealed a hydrodynamic diameter of approximately 4.5 nm for these particles. This approach represents a significant advancement in the field of nanotechnology applied to anticancer therapy. Au QCs not only provide an effective platform for controlled curcumin delivery but also offer the advantage of reducing the toxicity associated with free curcumin, making it a promising option for safer and more effective treatments. Additionally, good homogeneity in particle dispersion under TEM suggests that these C-Au QCs conjugates can be easily administered, allowing for a more even distribution in the body. This feature is crucial for maximizing the therapeutic efficacy of curcumin, especially in the context of cancer treatment.

### 3.2. Metal Oxide Nanoparticles

**Figure 3:**
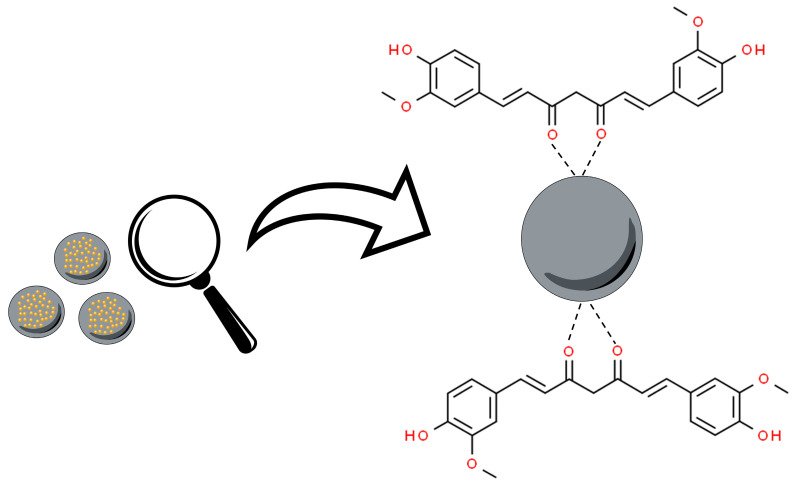
Overall scheme of curcumin’s oxygen atoms from the keto–enol groups’ interaction to metal oxide nanoparticles (MOx). Based on Bhandari et al., 2016 [63].

Metal oxide nanoparticles (MOx) are a fascinating class and diverse form of nanomaterials. These nanostructures are formed from metallic elements and oxygen under different conditions, generating different structural forms. Metal oxide nanoparticles have been associated with curcumin, from the interaction of the nanoparticle surface with the keto–enol functionality of this molecule (Figure 3) [63].

In this sense, Yallapu and partners developed magnetic metal oxide curcumin nanoparticles (MagNP-CUR) through the method of co-precipitation of Fe^2+^ and Fe^3+^ salts [64]. Magnetic nanoparticles (MagNPs) have been used because they are easy to prepare, chemically functional, have small sizes, excellent biocompatibility, stability, efficient drug conjugation, and remarkable magnetic capacity [65]. The production of iron-based MagNPs has been preferred compared to other metals, mainly due to biocompatibility and low toxic effects, which are essential due to the accumulation of the toxic effects of MNPs [66].

In this study, MagNP-CUR was characterized in terms of particle size and zeta potential by DLS. The nanoparticles had a particle size of 109 nm, compatible with the classification of superparamagnetic iron oxide nanoparticles (SPIONs), which are composed of iron particles with a size greater than 50 nm, with an average size between 50 and 100 nm [64]. It is noteworthy that the size of these particles and their superparamagnetic behavior are dependent on their crystalline structure and composition since the smaller the size, the lower the transition temperature from ferromagnetic to superparamagnetic behavior [67]. In addition, the decrease in particle size reflects a relative increase in surface effects. Further, temperature can influence the magnetic moment of each MNP to rotate randomly [66,68]. Finally, MagNP-CUC showed a neutral zeta potential charge of −0.99 mV [64]. Despite the low value of zeta potential, no aggregation of MNP-CUR was evidenced, justified by the properties of poly (propylene oxide) [64], whose chains confer a steric hindrance, preventing aggregation.

Sahne et al. developed graphene oxide nanoparticles (GO NPs) to encapsulate curcumin for anticancer treatment [22]. GO NPs have been considered an option in nanoformulations due to their ability to increase the bioavailability of bioactive compounds, the possibility of functionalization, and greater permeability and ability to increase cellular internalization. The authors developed GO NPs functionalized with carboxymethylcellulose (CMC) and folic acid (FA) and immobilized with poly (N-vinyl pyrrolidone) (PVP) using a layer-by-layer technique (Cur-FA-CMC/PVP GO NPs). Functionalization with CMC/PVP and FA did not change the morphology of the particles, which had a spherical shape with a hydrodynamic diameter of 60 nm and zeta potential of −48 mV due to its high concentrations of carboxylic and hydroxyl groups.

Kundu and collaborators produced inorganic nanoparticles made of Zn metal oxides [69]. Oxide metals, as ZnO, have been considered desirable options for nanoparticles due to their wide range of oxidation status, adjustable size and charge, biocompatibility, and ease of manipulation [70]. In this perspective, the authors developed nanoparticles of ZnO and ZnO-NH_2_, functionalizing them with 3-carboxybenzeneboronic acid (PBA) to vectorize curcumin to the tumor. The study did not disclose the nanoparticles production method. The particle sizes observed by DLS for ZnO NPs, ZnO-PBA, and ZnO-PBA-curcumin were 166.3 ± 7.9 nm, 284.96 ± 8.3 nm, and 413.63 ± 9.5 nm, respectively. The authors suggest that the apparent change in the size of the NPs was not significant, justifying the hydrodynamic diameter as an effect of the solvation of the nanoparticles.

On the other hand, in TEM analysis the size difference was smaller, wherein the values of ZnO NPs and ZnO-PBA-curcumin particle sizes were found to be between 30 and 40 nm [69]. XRD analysis of the peaks of ZnO NPs, ZnO-PBA, and ZnO-PBA-curcumin indicated a hexagonal crystalline structure. The zeta potential values for ZnO NPs, ZnO-PBA, and ZnO-PBA-curcumin were +17.9 ± 0.1 mV, −4.7 ± 0.31 mV, and −16.4 ± 0.30 mV, respectively [69]. The binding of negatively charged PBA molecules and the presence of curcumin, which shows several ionizable hydroxyl groups, explained the reverse charge observed in the zeta potential of positively charged ZnO NPs. According to Kundu and collaborators [69], curcumin was carried on the surface of ZnO-PBA NPs through ion–metal ligand coordination by forming a chelate ring with ZnO. Accordingly, the structure displayed a pH-dependent release of curcumin by the breaking down of the chelate complex, which is less stable at low pH [71]. Liu et al. [72] also observed a similar pH-dependent response in their zinc oxide NPs.

### 3.3. Mesoporous Silica Nanoparticles (MSNPs)

**Figure 4:**
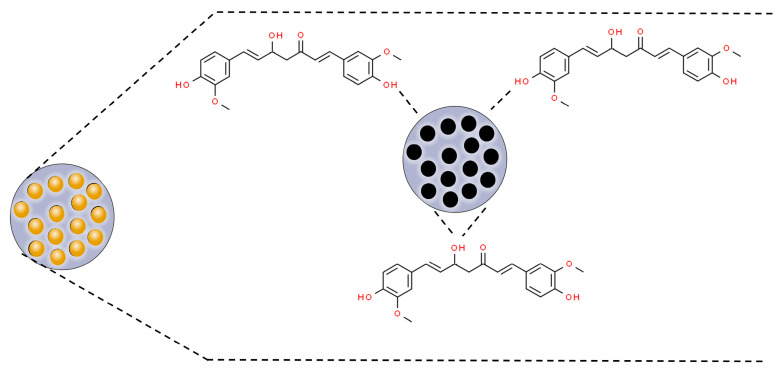
Scheme of curcumin incorporation to mesoporous nanoparticles’ (MSNPs’) pores by hydrogen bonds with SI-OH groups. Based on Jambhrunkar et al., 2014; Lungare et al., 2016; and Ribeiro et al., 2022 [79,80,81].

Mesoporous silica nanoparticles (MSNPs) are inorganic nanoparticles or mesoporous forms of silica with particle sizes between 30 and 300 nm that can promote endocytosis by target cells with minimal toxicity [73]. MSNPs are of special interest because of their excellent biocompatibility, high drug-loading capacity, rigid framework, well-defined pore structure, easily controllable morphology, and tunable surface chemistry [74,75]. The porous structure of mesoporous silica materials provides cavities that can host and release a great variety of biomolecules and therapeutic agents, such as anticancer drugs [76,77,78]. In this perspective, curcumin has been associated with MSNPs through hydrogen bonds with silica matrix groups (SI-OH) (Figure 4) [79,80,81].

Hence, Li and colleagues [82] produced curcumin-loaded redox-responsive mesoporous silica nanoparticles (MSNPs). The authors modified the mesoporous silica nanoparticles with thiol (MSNP-SS-COOH) and conjugated the surface with hyaluronic acid (HA) or polyethyleneimine–folic acid (PEI-FA) via disulfide bonds. MSNPs-HA and MSNPs-PEI-FA for curcumin delivery were evaluated against breast cancer in an animal model. MSNs were characterized in terms of particle size, zeta potential, morphology, and structure (SEM and TEM). The modifications made to the particle were evaluated by FTIR and UV-Vis. The TEM and SEM analyses revealed that MSNPs were spherical and porous. The structures of PEI-FA, MSNPs-PEI-FA, and MSNPs-HA were confirmed by FTIR spectroscopy and UV-Vis [82]. MSNPs-SS-COOH showed an increasing negative charge, according to the conjugation of HA. Upon PEI and PEI-FA conjugations, the surface charge changed from negative to positive, given the nature of these functionalizing agents. The surface load modifications confirm the functionalization made in the nanoparticles [82]. The size data revealed an increase in particles at each performed conjugation step, a behavior already reported in other studies [83].

### 3.4. Metal–Organic Frameworks (MOFs)

**Figure 5:**
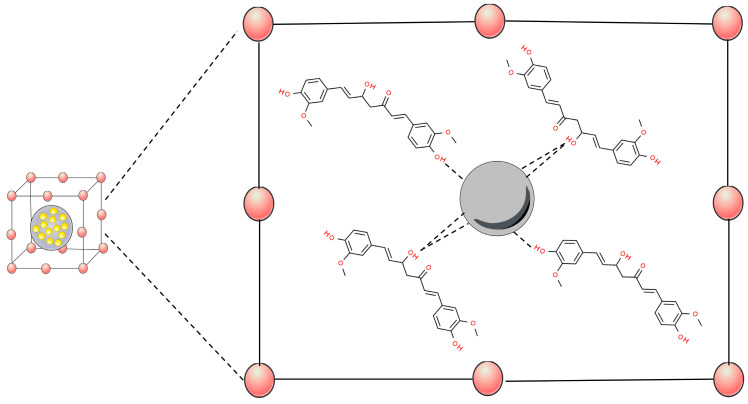
Scheme of metal–organic frameworks (MOFs)’ interactions of metals to curcumin hydroxyl groups. Based on Munasinghe et al., 2023 [88].

Metal–organic frameworks (MOFs) are a new type of crystalline porous material that is self-assembled by the coordination of metal cations/clusters with organic linkers [84,85]. MOFs have exhibited promising catalytic potentials towards many types of reactions, owing to their designable metal–oxo clusters bridging organic linkers, modifiable structure, and intrinsic porosities [86]. This nanomaterial has been widely developed as a carrier for the effective delivery of drugs to tumor tissues [87]. These systems have been associated with curcumin through interactions between the phenol and enol sites of this molecule, showing strong evidence of chelation-type bonds with the MOFs (Figure 5) [88].

Hence, Dehghani and colleagues developed nanoparticles with multifunctional theragnostic capacity. These derive from iron-based metal–organic frameworks (MOFs), called MIL-88B or simply MIL in this work, produced by hydrothermal synthesis reactions [89]. Curcumin was then loaded into the pores of MIL and folic acid chitosan conjugate (FC) was finally coated on the surface of the carrier to achieve cancer-specific targeting properties [89]. After loading curcumin in the MIL NPs, the zeta potential changed from −23.42 to −31.21 mV and further changed to −2.1 mV after the FC coating [89]. As revealed by SEM images, MIL-Cur-FC NPs are multi-faceted in shape, with an average width of 74 and length of 95 nm. TEM images of MIL and MIL-Cur-FC confirmed the presence of the FC layer on the surface of MIL. Curcumin loading and FC coating of MIL was confirmed by FTIR analysis and nuclear magnetic resonance spectrum showing an encapsulation efficiency of 98% [89].

Also working towards the development of MOFs, Laha and colleagues synthesized folic acid-conjugated curcumin-loaded nanoscale IRMOF-3 (IRMOF-3@CCM@FA) through a hydrothermal synthesis reaction [90]. IRMOF-3 belongs to a class of nanoscale MOFs (NMOFs) and falls under the category of inorganic–organic hybrid drug delivery systems. They are known for their effective encapsulation of chemotherapeutic agents and have gained relevance due to their large surface area, ultra-high tunable functionality, and porosity. The particle size of IRMOF-3 increased after the incorporation of curcumin (IRMOF-3@CCM) from 255.3 ± 7.99 to 342.7 ± 1.29 and further increased to 371.7 ± 8.80 with the incorporation of folic acid (IRMOF-3@CCM@FA). The zeta potential was low for particles without folic acid, −2.17 mV ± 0.15 for IRMOF-3 and −0.907 mV ± 0.01 for IRMOF-3@CCM. However, IRMOF-3@CCM@FA exhibited a zeta potential of −10.9 mV ± 0.50. Electron microscopy morphology analysis showed that IRMOF-3 nanoparticles had uniform spherical structures with a size of 75 ± 10 nm. By adding folic acid and curcumin, the IRMOF-3@CCM@FA shape was altered and the size increased to 117 ± 15 nm.

Another noteworthy subclass of MOFs is the zeolitic imidazolate frameworks (ZIFs). These materials offer several advantages, such as high porosity, tunable structure, large specific surface area, and exceptional chemical and thermal stability. Yu et al. synthesized curcumin-loaded ZIF-8 nanoparticles coated with hyaluronic acid (Cur@ZIF-8@HA) by a pH-assisted method for application in breast cancer treatment [91]. Through TEM and SEM analyses, the particles exhibited cubic morphology with sharp edges, truncated corners, and rough surfaces, with an average size of 184.1 ± 13.2 nm. The hydrodynamic diameter by DLS was 217.7 nm, with a PdI of 0.432. The zeta potential was −18.10 ± 1.08 mV. Finally, followed by curcumin´s quantification, the encapsulation efficiency was 9.6%.

Overall, inorganic nanoparticles are effective strategies for curcumin delivery. Different metals and oxide metals can be used to produce these nanoparticles, providing unique characteristics, and tuning the physicochemical properties of this molecule. In addition, these nanoparticles also allow functionalization of their surface, being effective for therapy and diagnosis in various types of cancer. Table 1 summarizes the composition, production method, and physicochemical characteristics of these nanoparticles.

## 4. Curcumin In Vivo Cancer Studies

Considering the previously mentioned pharmacological potential of curcumin as an anticancer agent, its biopharmaceutical limitations should be overcome for proper in vivo use [42,43]. Therefore, curcumin-based inorganic nanoparticles have been produced and investigated for their performance in different in vivo cancer models. In the sub-sections below, the biological impacts of different curcumin formulations are summarized to provide an overview of the potential of such curcumin-based formulations for prospective cancer studies. Additionally, a summary of in vivo data is expressed in Table 2.

### 4.1. Pancreatic Cancer

Pancreatic cancer (PC) remains one of the greatest challenges in oncology, with just over 11% of patients alive 5 years following diagnosis. Chemotherapy and surgery are the two main treatments for PC. Nevertheless, at diagnosis, only 15–20% of individuals are candidates for surgery [92]. Most PC patients have distant metastases when they are first diagnosed. As a result, excising the main lesion by surgical treatment is unlikely to improve the prognosis. Furthermore, PC is highly resistant to treatment [93]. To overcome this problem, curcumin can be a promising therapeutic alternative or complementary treatment, as this molecule efficiently prevents the growth of pancreatic cancer cells through a variety of pathways, including cell cycle rest at G2/M phase, induction of apoptosis, and autophagy [94].

Curcumin-loaded magnetic nanoparticles were developed, evaluated in vitro (cellular uptake, cell proliferation, and clonogenic assay), and in vivo in a PC model [64]. For the in vivo model, HPAF-II human pancreatic cancer cells were inoculated into male athymic nude (nu/nu) mice for 13 days. The animals were treated with 20 µg of curcumin dispersed in 100 µL of 0.1% Tween^®^ 20, while 20 µg of curcumin loaded in magnetic nanoparticles were dispersed in 100 µL PBS. Magnetic nanoparticles without curcumin and surfactant were evaluated to eliminate possible biases. Tumor size was monitored between 7 and 40 days. Immunohistochemistry and immunofluorescence of the tumor tissues were assessed. At the end of the treatment, curcumin-loaded metallic nanoparticles showed 71.2% of tumor inhibition, while free curcumin only inhibited 35.9%. The tumor increased over days in the control groups. Such an inhibitory profile of nanoparticles is related to the potentiation of the drug and the release modulation of curcumin by the system, increasing bioavailability for a larger period. Such hypotheses were corroborated by the immunohistochemically and immunofluorescence assays performed by the authors.

### 4.2. Lung Cancer

Lung cancer is a type of cancer that starts when abnormal cells grow out of control in the lungs. It is a health problem that can cause serious harm and it has one of the highest incidence rates of mortality [95,96]. Given this scenario, studies on molecules with anticancer potential have been conducted. Among these, curcumin appears to be an important candidate, as studies suggest that it inhibits the growth of lung cancer cells through multiple pathways, inducing apoptosis, and inhibiting cell proliferation and epigenetic changes [7,97].

In this perspective, Dehghani and colleagues prepared multifunctional metal–organic framework-based theragnostic nanoparticles targeting lung carcinoma cells [89]. The authors performed in vitro assays wherein cell compatibility and selective toxicity were observed. Additionally, in vivo studies were conducted by administering M109 cells (2 × 10^6^) subcutaneously in BALB/C mice. After the formation of tumors, the mice were treated by intravenous administration (5 mg/kg) with free curcumin, control (saline), curcumin nanoparticles (MIL-Cur NPs), and folic acid–chitosan–curcumin nanoparticles (FC-coated MIL-Cur NPs). The tumor sizes were monitored, and magnetic resonance imaging (MRI) was analyzed for uptake performance. In vivo MRI data showed high tumor uptake for MIL-Cur@FC, high T1–T2 contrast effect, and a decrease in tumor size after treatment.

### 4.3. Breast Cancer

Breast cancer is the most common malignant tumor in women in the world. It accounts for as much as 36% of oncological patients. Furthermore, its incidence is constantly increasing in all regions of the world [98]. The molecular characteristics of breast cancer are possible targets for therapies. These include the activation of human epidermal growth factor receptor 2 (HER2) and hormone receptors (estrogen receptor [ER] and progesterone receptor [PR]) [99]. Studies have shown that curcumin is capable of suppressing breast cancer by altering the amounts of receptors (Her-2 receptor, IR, ER-a, and Fas) and growth factors (PDGF, TGF, FGF, and FEG) [100,101,102].

Given curcumin’s anti-breast cancer potential and the damage caused to tumor cells by inorganic nanoparticles, curcumin-based metal oxide nanoparticles were developed and their performance against breast cancer was evaluated [69]. Phenylboronic acid-functionalized ZnO nanoparticles (PBA-CURC-ZnO-NC) were synthesized. Initially, researchers evaluated the impact of these nanoparticles on cellular viability, intracellular reactive oxygen species, potential mitochondrial membrane, and cell death examination in an in vitro perspective to better assess the mechanism of action of this system against cancer cells. Subsequently, the effect of this system in an in vivo solid tumor model was assessed. To this end, Ehrlich ascites carcinoma (EAC) was injected into mice for tumor development. PBA-CURC-ZnO-NCs showed pronounced anticancer activity compared to empty nanoparticles and free curcumin. However, in terms of tumor volume alone free curcumin and PBA-CURC-ZnO-NCs showed no significant changes. The empty nanoparticles and untreated groups exhibited tumor growth at the end of 14 days [69]. Regarding tissue morphology, the rupture and cellular invasion in the tissues of the untreated tumor-bearing mice were noticeable [69]. Contrariwise, the tissues of mice treated with PBA-CURC-ZnO-NCs showed restoration and reduction in tumor cell infiltration. Further, an increase in caspases-9 and cleaved caspases-3 was observed [69]. Apoptosis promoted by curcumin-loaded nanoparticles could be suggested due to an increase in caspases, causing a reduction in breast cancer cells [103,104].

Taking a different formulation route, Mahalunkar et al. studied functionalized folate–curcumin-loaded gold–polyvinylpyrrolidone (FA-Curc-AU-PVP) nanoparticles for targeted delivery in breast cancer models [50]. In vitro tests such as viability and cell cycle investigation were performed. The in vivo approach of this study involved tumors stimulated from 4T1 mammary carcinoma injection in female BALB/C mice. After tumor growth, mice were treated by intratumoral administration (10 mg/kg) of free and nano-encapsulated curcumin (FA-Curc-AU-PVP nanoparticles) for 2 weeks. Tumor volume was used as an antitumor efficacy parameter. No significant decrease in tumor volume was observed after free curcumin treatment; however, FA-Curc-AU-PVP nanoparticles significantly inhibited tumor growth. The above data suggest that FA-Curc-AU-PVP nanoparticles increased the efficacy of curcumin at a dose as low as 10 mg/kg body weight. Such responses were attributed to the functionalization of nanoparticles, which allows recognition in folic acid receptors, promoting tumor targeting and consequently drug potentiation [50].

In the same pathway, Sahne and colleagues [22] developed graphene oxide nanoparticles with a single layer of CMC conjugated to PVP and functionalized with folic acid (Cur-FA-CMC/PVP GP NPs). The researchers assessed the in vivo effectiveness of these particles in treating breast cancer. To conduct the study, they induced a 4T1 tumor model in BALB/C mice and administered 200 μL of Cur-FA-CMC/PVP GO NPs (equivalent to a curcumin dose of 4 mg/kg). They examined drug uptake in tumor cells using immunohistochemistry analysis and assessed the antitumor efficacy by monitoring daily changes in tumor size and body weight. Upon completion of the study, they examined the tumor to assess apoptosis, tumor blood vessels, and inflammatory response. The results revealed a notable concentration of particles at the tumor site, a favorable in vivo pharmacokinetic profile, and an effective tumor targeting and accumulation. The accumulation of particles induced apoptosis, leading to the inhibition of tumor growth. Upon evaluating the tumor weight, a remarkable tumor growth inhibition rate of 76% was observed. Consequently, this study demonstrated that the developed system holds promise as a carrier for curcumin, exhibiting significant potential for anti-breast cancer activity [22].

Khandelwal and colleagues also worked on gold nanoparticles like the studies cited previously in this paper; however, they used quantum clusters. The developed curcumin-conjugated Au quantum clusters (C-Au QCs) were assessed against breast cancer models both in vitro and in vivo [62]. After assessing the inhibitory potential of C-Au QCs on the growth of MCF7 breast cancer cells in vitro, they conducted an in vivo study using a murine xenograft model to evaluate the impact of these nanoparticles on tumor growth. Following the induction of tumors, 20 mg/kg of C-Au QCs were administered intraperitoneally three times a week. Four weeks later, it became evident that the size, volume, and weight of the tumors had significantly decreased in the group treated with the nanoparticles compared to the control group, demonstrating the promising potential of this system for cancer treatment.

Also aiming at breast cancer, Laha and colleagues developed curcumin nanoparticles in an isoreticular nanoscale metal–organic framework (NMOF-3) and evaluated tumor targeting in a mouse model [90]. The researchers evaluated the antitumoral activity of curcumin-loaded IRMOF-3 (IRMOFR-3@CCM) and folic acid-conjugated curcumin-loaded IRMOF-3 (IRMOF-3@CCM@FA). The tumors were allowed to develop for ten days. Subsequently, IRMOF-3, IRMOF-3@CCM, and IRMOF-3@CCM@FA were administered to triple-negative tumor-bearing BALB/C mice over 25 days. Tumor volume in the mice was measured for up to 30 days. They observed that the groups treated with IRMOF-3@CCM and IRMOF-3@CCM@FA showed significant tumor suppression compared to the untreated group. On day 30, the average tumor volume for the control, IRMOF-3-, IRMOF-3@CCM-, and IRMOF-3@CCM@FA-treated mice was 0.86 mm^3^, 0.756 mm^3^, 0.479 mm^3^, and 0.332 mm^3^, respectively. On day 30, the average tumor weight for the untreated, IRMOF-3-, IRMOF-3@CCM-, and IRMOF-3@CCM@FA-treated mice was 451.33 mg, 278.13 mg, 115.8 mg, and 67.26 mg, respectively. This work evidenced that the active targeting of the tumor by the developed nanostructure greatly decreased tumor size when compared to unfunctionalized (non-vectorized delivery) formulations.

Yu and colleagues developed zeolitic imidazolate framework-8 nanoparticles coated with hyaluronic acid (HA) to deliver curcumin (Cur@ZIF-8@HA) [91]. These particles were assessed against an in vivo breast cancer model. Three groups of mice with tumors were treated with saline, Cur@ZIF-8, and Cur@ZIF-8@HA. After ten days of treatment, they observed that the groups treated with saline and Cur@ZIF-8 exhibited a significant weight reduction, indicating that Cur@ZIF-8@HA mitigates systemic toxicity during treatment. Furthermore, they observed fewer variations in tumor volumes within the group treated with Cur@ZIF-8@HA, suggesting the higher antitumoral effectiveness of the formulation functionalized with hyaluronic acid. In addition, the researchers assessed the in vivo antimetastatic effect of Cur@CIF-8 and Cur@ZIF-8@HA. The tumor model used for the treatment, the 4T1 orthotropic mammary tumor, has been employed to investigate spontaneous metastatic cancer, encompassing all metastasis steps and visible metastatic nodules in the lungs. The results indicated that mice treated with Cur@ZIF-8 exhibited extensive tumor burdens in the excised lungs, whereas Cur@CIF-8@HA displayed a lower tumor burden. These results suggest that HA enhanced the delivery of the nanoparticles to the tumor cells, reducing systemic toxicological effects and enhancing their inhibitory potential against pulmonary metastasis.

Leaning beyond metal structures, Li and colleagues evaluated the antitumor efficacy of curcumin-loaded mesoporous silica nanoparticles (MSNPs) functionalized with polyethyleneimine–folic acid (PEI-FA) in a breast cancer cell line and a mouse xenograft model [82]. A 0.1 mL dispersion of MDA-MB-231 cells was administered subcutaneously in female BALB/C nude mice. After reaching a 4 mm tumor, 8 mg/kg free curcumin and mesoporous silica nanoparticles functionalized with PEI-FA were injected by the tail every three days. The authors observed that the nanoparticles were internalized by MDA-MB-231 cells releasing curcumin at the target site. Inhibition of tumor growth was also noticeable, wherein the treated tumors with curcumin-loaded mesoporous silica nanoparticles functionalized with polyethyleneimine–folic acid were smaller than those treated with free curcumin alone. Finally, no effects suggesting the toxicity of this formulation in mice were observed [82].

### 4.4. Colorectal Cancer

Colorectal cancer (CRC) is a disease that is highly prevalent and recurrent. This cancer accounts for approximately 10% of all cancer cases and is the second leading cause of cancer-related deaths worldwide [105]. Furthermore, it is estimated that colorectal cancer will increase by 60% in 2030, leading to a mortality rate of over 1.1 million [106].

Given this scenario, molecules with anticancer potential such as curcumin have been investigated. Curcumin can interrupt the cell cycle, in addition to accelerating cell death, inhibiting the spread of colorectal cancer [107].

Accordingly, Alibolandi et al. developed a curcumin theragnostic nanomedicine [55]. Theragnostic merges the ability to target and deliver therapeutic agents with imaging for diagnostic purposes. Nanomaterials such as gold and iron oxide nanoparticles are often studied for these purposes [108]. In this study, in vitro cytotoxicity, in vivo cytotoxicity, and CT scanning assays were performed to evaluate Apt-PEG-AuPAMAM-CUR efficiency as a theragnostic agent [55]. First, to assess toxicity and the impact of MUC-1 aptamer, two cell lines MUC-1 positive (HT29 and C26, human and murine colon cancer) and one cell line MUC-1 negative (CHO, Chinese hamster ovary) were used. Further, a C26 tumor-bearing mice model was used for anticancer activity. After 14 days of subcutaneous inoculation, mice were treated intravenously with free curcumin, PEG-AuPAMAM-CUR, Apt-PEG-AuPAMAM-CUR (curcumin equivalent to 2 mg/kg), or saline 0.9% as negative control twice per week for three weeks.

Additionally, CT scans were performed with animals treated with Au-NP, PEG-AuPAMAM-CUR, and Apt-PEG-AuPAMAM-CUR. The antitumor activity of PEG-AuPAMAM-CUR was higher than free curcumin. This effect can be attributed to the EPR effect after intravenous injection, wherein nanoparticles are rapidly distributed into the body and accumulated into the tumor site via fenestrated blood vessels. Additionally, Apt-PEG-AuPAMAM-CUR significantly increased curcumin’s cancer cell toxicity by binding to MUC-1 cancer cells, which enhances receptor-mediated endocytosis. Those in vivo results are supported by in vitro experiments, wherein dendrimers were selective to MUC-1 positive cell lines (C26 and HT29) and further supported by CT scans, which showed increased intensity at the tumor site after treatment with Apt-PEG-AuPAMAM-CUR [55].

## 5. Limitations and Future Directions

The formulations cited in this study are inorganic nanoparticles, many of which are functionalized by organic molecules. Although relevant for the future direction of cancer treatment, the complexity of these formulations is likely to increase the cost of production, so the value of the final nanotechnological product is expected to be high, as is the value of many nanostructured products available in the market. Therefore, the cost of research and production is one of the main limitations when it comes to these types of formulations. Hence, although curcumin-containing inorganic-based formulations reach the market, overall prices are prone to be high. On the other hand, proper pharmacoeconomics should be addressed in due time, once these formulations might reduce the number of interventions, time of hospital stay, and associated treatment costs.

By conducting this review, it was possible to observe an absence of detailed information, which limits reproducibility, proper interpretation, and fast advance of science. For instance, a few studies did not disclose information on formulation concentration (mg/mL) and dosage (mg/kg). Further, others did not state whether the administration followed a maximum volume per kilogram of animal. Furthermore, different concentrations of curcumin in different nanoparticle vehicles, as well as not using groups with drug-free nanoparticles, including functionalized ones, created a bias in evaluating effectiveness, since different compositions can generate unknown physiological effects.

In addition, only a few studies have performed curcumin pharmacokinetic tests, which may limit the understanding of the nanostructures’ efficacy. Finally, only a few studies have evaluated toxicity-related symptomatology, demonstrated by lethargy, vomiting, diarrhea, and elevated body temperature, as these are symptoms that are relevant after administration of single-dose or multi-dose chemotherapy agents administered intravenously. Despite the aforementioned limitations, the outlook is for studies containing inorganic nanoparticle formulations to become increasingly robust, with detailed information on their development, characterization, and pre-clinical assays.

Despite the promising results that encourage curcumin inorganic nanoparticles, there are still numerous challenges for these nanosystems to be introduced into the market. More robust efficacy and safety studies in different pre-clinical and clinical models need to be conducted before approval by global drug regulatory agencies. It is also hoped that production costs can decrease over time, facilitating the scaling up of this type of formulation and increasing the interest in these nanoparticles, prompting the transfer of these technologies from benchtop to bedside.

## 6. Final Considerations

Natural products have always made contributions to medicine, and it is no different in cancer therapy. Natural compounds such as polyphenols have been linked to anticancer potential. Curcumin is a polyphenol molecule of natural origin with high anticancer therapeutic potential. However, its biopharmaceutical limitations may compromise its therapeutic efficacy. Given this drawback, this review showed that this molecule has been associated with nanoparticles, a promising strategy for increasing the apparent solubility, stability, targeting, permeability enhancement, and selectivity for cancer cells. Among the universe of nanoparticles, inorganic nanoparticles stand out, which have optimized physicochemical properties when compared to the organic ones commonly used in recent years.

In addition to being more stable, inorganic nanoparticles present an anticancer intrinsic potential that, combined with therapeutic molecules such as curcumin, may generate a promising therapy in the treatment of this disease. Thus, this review compiled recent advances in inorganic nanoparticles intended for cancer therapy, evaluated in different in vivo models. From the available studies, it was possible to observe that overall sizes around 250 nm or below appear most desirable. Overall, it was possible to demonstrate that the association of curcumin with the different inorganic nanoparticles presented revealed increased anticancer activity when compared to this molecule in its free form. In addition, the studies demonstrated that active vectorization, associated with passive vectorization by EPR effect, is desirable to increase the in vivo activity of curcumin. From the assessed models, we should consider that breast cancer was the most studied cancer type. This suggests that the other observed cancer types could be further investigated regarding curcumin inorganic nanoparticles. In addition, future studies are still required in other models, such as skin cancers, lymphomas, leukemias, and other critical cancers.

## Figures and Tables

**Table 1:** Curcumin inorganic nanoparticles’ composition, production method, and physicochemical characteristics.

Nanoparticles	Composition	Method	Physicochemical Characteristics	References
MNPs	FA-Au-PVP	LBL assembly	Size: 72–350 nm and zeta potential: −9 mV	[50]
MNPs	PEG-AuPAMAM	Synthesis and dialysis	Size: 5 nm, zeta potential: ~4 mV, and EE: 88%	[55]
MNPs	Au QCs	Synthesis	Size: ~2.3–~20.2 nm	[62]
MOx	Iron	Co-precipitation	Size: ~100 nm and zeta potential: −0.99 mV	[64]
MOx	CMC, PVP, graphene oxide	Synthesis, acetylation, and polymerization	Size: 60 nm, zeta potential: −48 mV, EE: 94%, and DLC: 47%	[22]
MOx	PBA–ZnO	Precipitation	Size (TEM): 30–40 nm and zeta potential: −16 mV	[69]
MSNPs	Silica, polyethyleneimine, folic acid or hyaluronic acid	Synthesis	Size: 90 nm	[82]
MOFs	Iron–MOF	Hydrothermal synthesis reaction	Size: ~100 nm, zeta potential: −30 mV, and EE: 98%	[89]
MOFs	IRMOF-3, folic acid	Synthesis and precipitation	Size: 255.3–371.7 nm, zeta potential: −0.907 to −10.09 mV, EE: 98%, and DLC: 52%	[90]
MOFs	ZIF-8 and hyaluronic acid	Synthesis and precipitation	Size: 184.1 nm and DLC: ~10%	[91]

MOx: metal oxide nanoparticles; MNPs: metal nanoparticles; MSNPs: mesosporous silica nanoparticles; MOFs: metal–organic frameworks; PBA-ZnO: phenylboronic acid-conjugated zinc oxide; FA-Au-PVP: folic acid-conjugated gold–polyvinylpyrrolidone; PEG-AuPAMAM: polyethylene glycol gold–polyamidoamine; Au QCs: gold quantum clusters; IRMOF-3: isoreticular metal–organic framework-3; ZIF-8: zeolitic imidazolate framework; CMC: carboxymethyl cellulose; PVP: polyvinylpyrrolidone; LBL: layer-by-layer; EE: encapsulation efficiency; TEM: transmission electron microscopy; DLC: drug-loading content.

**Table 2:** Curcumin inorganic nanoparticles’ anticancer performance in vivo.

Cancer	Nanoparticles	Administration Regimen	In Vivo Model	In Vivo Performance	References
Pancreatic	MNP	20 µg/100 mL for 40 days	HPAF-II human pancreatic cancer cells inoculated into male athymic nude (nu/nu) mice	Increase in tumor inhibition	[64]
Lung	FC-coated MIL	Intravenous (5 mg/kg)	Subcutaneously M109 cells (2 × 10^6^) in Balb/C mice	Decrease in tumor size; high uptake	[89]
Breast	PBA-ZnO	Intravenous for 14 days every other day (10 mg/kg)	Ehrlich ascites carcinoma was injected into mice (107 cells per 50 mL/mouse)	Decrease in tumor; increase in caspases-9 and caspases-3 cleaved apoptosis induction mechanism	[69]
Breast	FA-Au-PVP	Intratumoral per 2 weeks (10 mg/kg)	4T1 mammary carcinoma injection in female BALB/C mice	Tumor growth was inhibited	[50]
Breast	Au QCs	Breast cancer cells injected subcutaneously into the flank areas (20 mg/kg)	Mouse xenograft model MDA-MB-231 cells female BALB/C nude mice	Tumor growth was inhibited	[62]
Breast	Cur-FA-CMC/PVP GO NPs	Subcutaneous injection via tail (200 µL, 4 mg of curcumin/kg)	4T1 breast cancer model in BALB/C mice	Tumor volume supression; tumor weight decrease	[22]
Breast	IRMOF-3 FA	100 µL injected subcutaneously into mammary	Triple negative tumor-bearing BALB/C model	Tumor volume supression; tumor weight decrease	[90]
Breast	ZIF-8-HA	Subcutaneous injection	4T1 xenograft model in BALB/C mice	Tumor growth inhibited; tumor weight decrease	[91]
Breast	PEI-FA MSN	Intravenous by the tail every three days (8 mg/kg)	Mouse xenograft model MDA-MB-231 cells female BALB/C nude mice	Tumor growth was inhibited	[82]
Colorectal	Apt-PEG-AuPAMAM	Intravenous per week for three weeks (2 mg/kg)	C26 tumor-bearing mice model was used for anticancer activity after 14 days of subcutaneous inoculation	Higher cellular uptake; higher cytotoxicity	[55]

PBA-ZnO: phenylboronic acid-conjugated zinc oxide; PLGA-PEG: poly lactic acid-co-glycolic acid–polyethylene glycol; FA-Au-PVP: folic acid-conjugated gold–polyvinylpyrrolidone; PEI-FA MSN: polyethyleneimine–folic acid mesoporous silica nanoparticles; Au QCs: gold quantum clusters; FA-CMC/PVP GO NPs: folic acid-conjugated carboxymethyl cellulose/polyvinylpyrrolidone graphene oxide nanoparticles; IRMOF-3 FA: isoreticular metal–organic framework-3 folic acid conjugated; ZIF-8 HA: hyaluronic acid-coated zeolitic imidazolate framework; MNP: metal nanoparticle; Apt-PEG-AuPAMAM: aptamer–polyethylene glycol gold–polyamidoamine; FC: folic acid–chitosan conjugated.

## Data Availability

Not applicable.
